# Noncovalent Interactions and Crystal Structure Prediction of Energetic Materials

**DOI:** 10.3390/molecules27123755

**Published:** 2022-06-10

**Authors:** Yan Liu, Chongwei An, Ning Liu, Minchang Wang, Baoyun Ye, Dongjie Liao

**Affiliations:** 1 School of Environment and Safety Engineering, North University of China, Taiyuan 030051, China; 18234159991@sina.cn (Y.L.); 18334788650@163.com (B.Y.); liaodongjie163@163.com (D.L.); 2 Department of Environmental and Safety Engineering, Taiyuan Institute of Technology, Taiyuan 030008, China; 3 Xi’an Modern Chemistry Research Institute, Xi’an 710065, China; flackliu@sina.com (N.L.); wmc204@163.com (M.W.)

**Keywords:** energetic material, molecular dynamic, crystal, energy, intermolecular interaction

## Abstract

The crystal and molecular structures, intermolecular interactions, and energy of CL-20, HATO, and FOX-7 were comparatively predicted based on molecular dynamic (MD) simulations. By comparison, the 2D fingerprint plot, Hirshfeld surface, reduced density gradient isosurface, and electrostatic potential surface were studied to detect the intermolecular interactions. Meanwhile, the effects of vacuum and different solvents on the crystal habit of CL-20, HATO, and FOX-7 were studied by AE and MAE model, respectively. The energy calculation was also analysed based on the equilibrium structures of these crystal models by MD simulations. Our results would provide fundamental insights for the crystal engineering of energetic materials.

## 1. Introduction

Energetic materials (EMs), mainly including explosives, propellants, and pyrotechnics are a kind of important compounds that can store and release considerable energy, and have occupied an important place in mining, military equipment, space exploration, and fireworks [1,2,3,4,5,6].

The 2,4,6,8,10,12-Hexanitro-2,4,6,8,10,12-hexaazaisowurtzitane (CL-20) with a cage-shaped molecular structure is currently the most powerful and commercially available EM [7]. Though the CL-20 is the highest energy-density explosive than traditional EMS, it has failed to meet widespread application in many weapons due to its higher impact sensitivity and poorer security property [8].

Dihydroxylammonium 5,5′-bistetrazole-1,1′-diolate (HATO or TKX-50) is a newly synthesized explosive with excellent comprehensive properties, which possesses lower impact sensitivity and higher detonation performance than the common explosives. Apart from the higher energy level and lower sensitivity, low toxicity is another advantage of HATO. However, poor crystal habit, irregular crystal morphology, and smaller crystal particle make an extremely constraint in its application [9,10,11].

The 1,1-diamino-2,2-dinitroethene (FOX-7) is synthesized in 1998 and is a push–pull symmetrical structure. There is a nitro-enamine structure with two nitro group at one carbon and two amino group at other in its molecular structure. Due to the special chemistry structure, FOX-7 possesses an excellent detonation performances and safety property, and is honoured as outstanding low sensitivity and highly energetic materials. However, poor crystal quality, monotonous crystal particle, more crystal defect, and poor solubility make its widespread application enormous challenging [12,13,14,15].

To meet the high requirement, the study on Ems with high crystal quality has always been an ultimate goal that researchers strive for. The morphology of energetic materials is a vital component and plays an important role in the crystal quality. It can be affected by intermolecular interaction, solvent, additive, and temperature in the process of crystallization [16,17]. Nevertheless, the crystallization mechanism of CL-20, HATO, and FOX-7 remain unclear, which significantly motivates us to probe the crystal habit in three explosives.

Herein, CL-20, HATO, and FOX-7 were chosen for comparatively simulating their structures and predicting their properties by quantum chemistry calculation. By comparison, the intermolecular interactions of CL-20, HATO, and FOX-7 were studied by two-dimensional (2D) fingerprint plots, Hirshfeld surfaces, reduced density gradient (RDG) isosurface, and electrostatic potential surface analysis. Then, the effects of different solvents and additives on the crystal structures of CL-20, HATO, and FOX-7 were evaluated. This work aims to deepen the understanding of crystal growth and provide a better insight for the crystallization process of energetic material.

**Figure 1:**
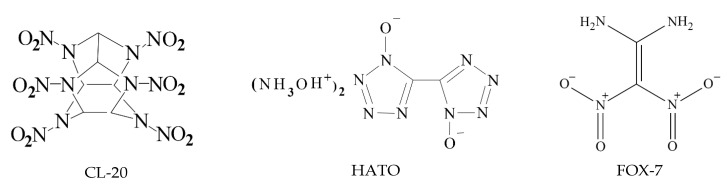
The chemical diagrams of CL-20, HATO, and FOX-7, respectively.

The chemical diagrams of CL-20, HATO, and FOX-7 are shown in Figure 1.

## 2. Results and Discussion

### 2.1. Hirshfeld Surface Analysis

**Figure 2:**
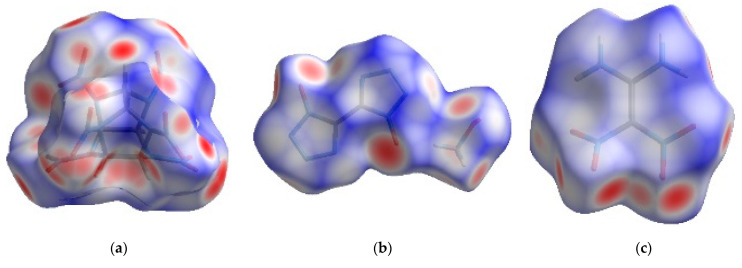

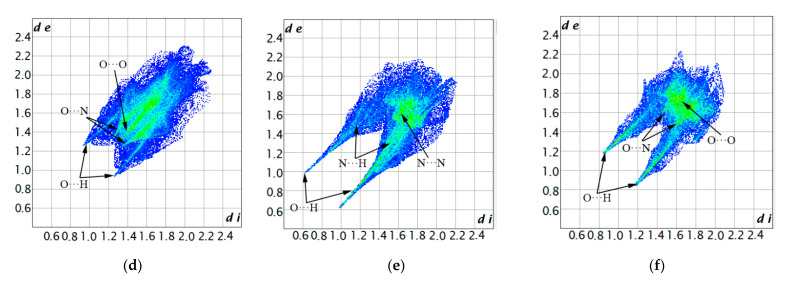
**The** 2D fingerprints plots and the associated Hirshfeld surfaces: Images (**a**–**c**) showing the 2D fingerprints of CL-20, HATO, and FOX-7, respectively; Images (**d**–**f**) showing the Hirshfeld surfaces of CL-20, HATO, and FOX-7, respectively.

The surface and 2D fingerprint plot, which was formed by the location of (*d*_i_ *d*_e_) point and their relative frequency, can confirm distance and intensity of intermolecular interaction [18,19]. The 2D fingerprints and the associated Hirshfeld surfaces of CL-20, HATO and FOX-7 were shown in Figure 2, in which the intermolecular interaction was visualized.

As shown in Figure 2a–c, there are the Hirshfeld surfaces of CL-20, HATO and FOX-7, respectively. The shape of surface of HATO and FOX-7 is plate, which indicates an insensitivity energetic material than CL-20. Furthermore, there are red, white, and blue dots on the surface, which represents the close contact around molecules. The red dot represents the distance *d* less the Van der Waals, white dot represents equal and blue dot represents exceeds. Compared with HATO and FOX-7, more red dots, which represent the predominant intermolecular interaction, are located on the surface of CL-20, particularly at edges, indicating that high close contact around CL-20 molecule.

In Figure 2d–f, we can see the intermolecular interaction in 2D fingerprints plots of crystals of CL-20, HATO, and FOX-7, respectively. We can see that there are O···H, O···N, and O···O intermolecular interactions in both of CL-20 and FOX-7 crystal. Compared with CL-20 and FOX-7, intermolecular interactions are O···H, O···N, and O···O in HATO. In addition, we can know that there are O···H intermolecular interactions in all of CL-20, HATO, and FOX-7, and it is a pair of remarkable spikes on the bottom left in 2D fingerprints.

### 2.2. Reduced Density Gradient Analysis

**Figure 3:**
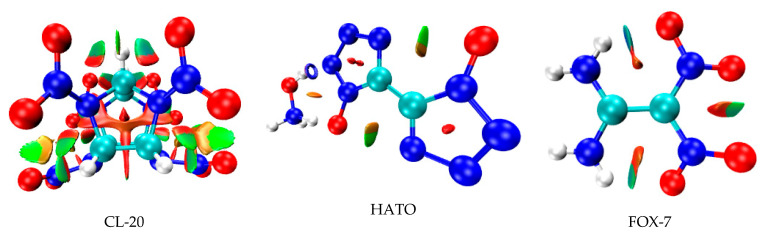
NCI plots of gradient isosurfaces.

To further reveal the information on inter- and intra-molecular effects and comprehensively study their influence on crystal structure, on the basis of the electron density and its RDG, the NCI plots of CL-20, HATO, and FOX-7 were determined in real space. In this method, the differences between hydrogen bonds, Van der Waals interactions, and repulsive steric clashes were observed by the study of the relationship between quantum-mechanical electron density and the reduced density gradient [20,21,22,23]. As shown in Figure 3, it is clear that there are abundant elliptical green slabs around the nitro group of each molecular edge, which denotes that weak hydrogen bonds and strong Van der Waals interactions exist in CL-20, HATO, and FOX-7. In addition to this, we can see that there are red slabs among five-membered ring of CL-20 and HATO, which indicates the steric effect in CL-20 and HATO. Due to more red slabs, CL-20 possess more greater steric effect than HATO. Due to red region only on the edge of elliptical slabs, FOX-7 possess the weakest steric effect in three molecule structure.

### 2.3. Electrostatic Potential Surface Analysis

**Figure 4:**
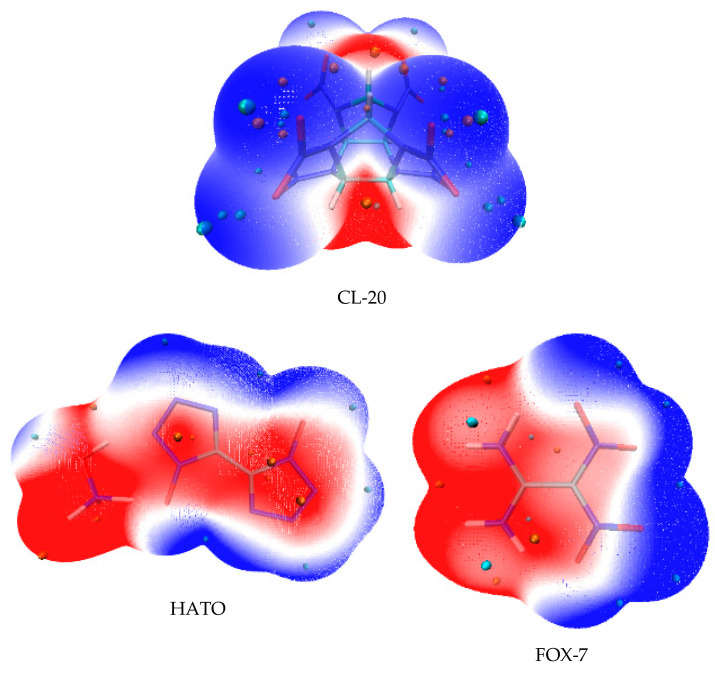
Electrostatic potential surfaces.

Electrostatic potential surfaces (ESP) illustrate the necessary energy that a unit positive charge moves from infinity to a certain position in a chemical system [24]. It clearly shows the charge density distribution and the charge variation region of molecule, which can be used to determine the electrophilicity and nucleophilicity at different positions in the system, and also to reveal the properties of non-covalent molecular interaction [25,26]. Hence, the ESP was carried out in order to understand the electronic property and the bond strength variation in CL-20, HATO, and FOX-7. The ESP mapped surfaces of CL-20, HATO, and FOX-7 were presented in Figure 4.

In Figure 4, it can be seen that the surface regions of ESP are coloured by different colours and the sites of maxima and minima are depicted by orange and cyan spheres, respectively. The blue region indicates negative ESP, which is the attraction of the proton by the concentrated electron density [27,28]. The blue region unexceptionally distributes on the edges of the molecules. Particularly, it situates on -NO_2_ group (in CL-20 and FOX-7) and -NO group (in HATO). The red region indicates positive ESP, which is the repulsion of the proton by the atomic nuclei in regions where low electron density exists and the nuclear charge is incompletely shielded [27,28]. The red region distributes on the edge of the -NH group (in HATO and FOX-7) and the central regions of the CL-20 molecule. When the polar of ESP is the same on the solvent and crystal face, they are manifested as a repulsive interaction, while the attractive interaction exists between the solvent and crystal face, when they are the opposite polar. The ESP qualitatively expresses the charged areas of molecules and the site of molecular reactively [29].

### 2.4. Crystal Morphology in Vacuum and Dominant Crystal Facets Analysis

**Figure 5:**
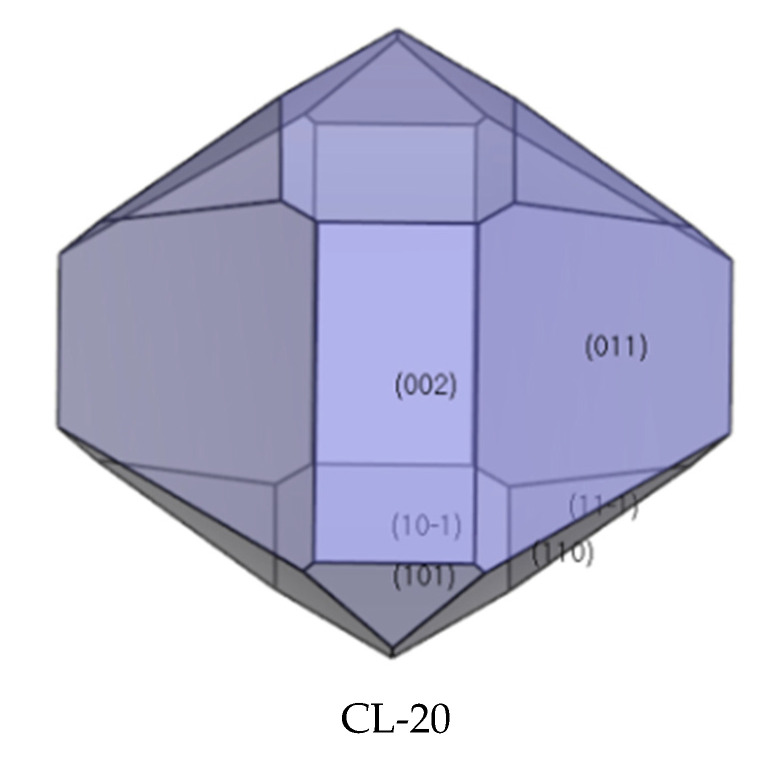

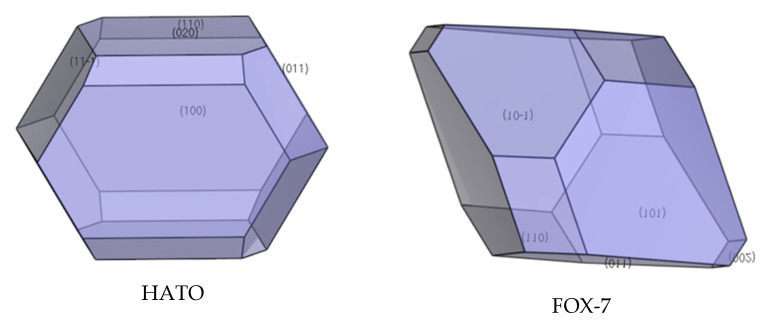
Crystal growth morphology in vacuum.

**Figure 6:**
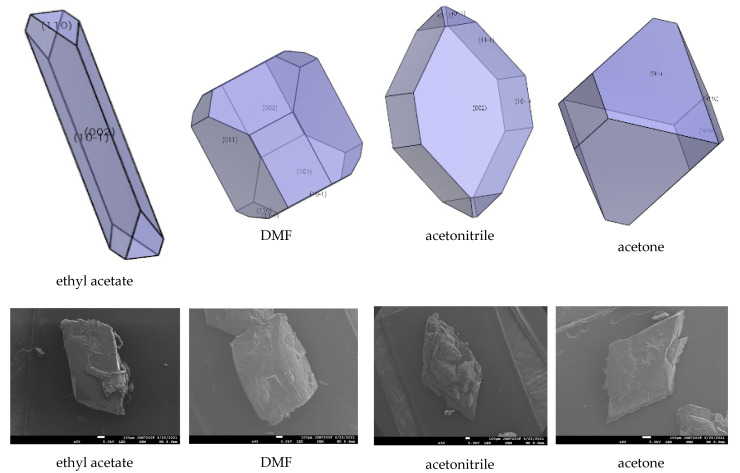
Crystal growth morphology of CL-20 in solvent by MAE model and recrystallization experiment.

The morphologically important facets of CL-20 crystal were simulated by the growth morphology algorithm (see Figure 5 and Table 1). This process was calculated based on the AE model, which was proposed by Hartman and Bennema according to the PBC theory in 1980. The attachment energy (*E*_att_) describes an amount of energy release when a crystal slice of thickness *d*_hkl_ attached to the (*hkl)* facet, and this model does not consider the solvent media, temperature, pressure, and other factors during the simulation process. It can be seen from Figure 6 that the aspect ratio of CL-20 is 1.44 and there are six dominant crystal facets in CL-20, including {0 1 1}, {1 0 −1}, {1 1 0}, {1 1 −1}, {0 0 2}, and {1 0 1}. It can be seen from Table 1 that the largest area percentage is {0 1 1} facet, so this facet exhibits the most morphological important in CL-20 crystal morphology. Due to a larger interplanar spacing {*d*_hkl_}, {0 1 1} and {1 0 −1} facets are the slow-growing facets whereas other facets occupy smaller {*d*_hkl_} are the fast-growing facets.

The morphologically important facets of HATO (see Figure 5, Table 2) and FOX-7 (see Figure 5, Table 3) crystals can be seen from the below figures and tables. In Figure 6, we can know that with an aspect ratio of 1.88, the predicted morphology of HATO crystal is mainly dominated by the {1 0 0}, {1 1 0}, {0 2 0}, {0 1 1}, and {1 1 −1} crystal facets. In Table 2, the largest area percentage of HATO is (1 0 0) facet, and this facet is the most morphological important in HATO crystal morphology. With a larger interplanar spacing {*d*_hkl_}, {1 0 0} facet is the slow-growing facet whereas other facets occupy smaller {*d*_hkl_} are the fast-growing facets. In the same way, the aspect ratio of FOX-7 is 1.83, and the dominant crystal facets of FOX-7 crystal are the {0 1 1}, {1 0 −1}, {0 0 2}, {1 0 1}, and {1 1 0} crystal facets (shown in Figure 6). In Table 3, the largest area percentage of FOX-7 is {0 1 1} facet, and this facet is the most morphological important in FOX-7 crystal morphology. With a larger interplanar spacing {*d*_hkl_}, {1 1 0} facet is the slow-growing facet whereas other facets occupy smaller {*d*_hkl_} are the fast-growing facets.

Apart from, there is *E*_att_ mainly composed with electrostatic interaction, Van der Waals force and hydrogen bonding interaction in three crystals. In CL-20 and FOX-7, the *E*_att_ is mainly involved Van der Waals force and electrostatic interaction, whereas, in HATO, the *E*_att_ is mainly involved in Van der Waals force and hydrogen bonding interaction.

### 2.5. Effect of Ethyl Acetate, Acetonitrile, Acetone, DMF, DMSO, and NMP

**Figure 7:**
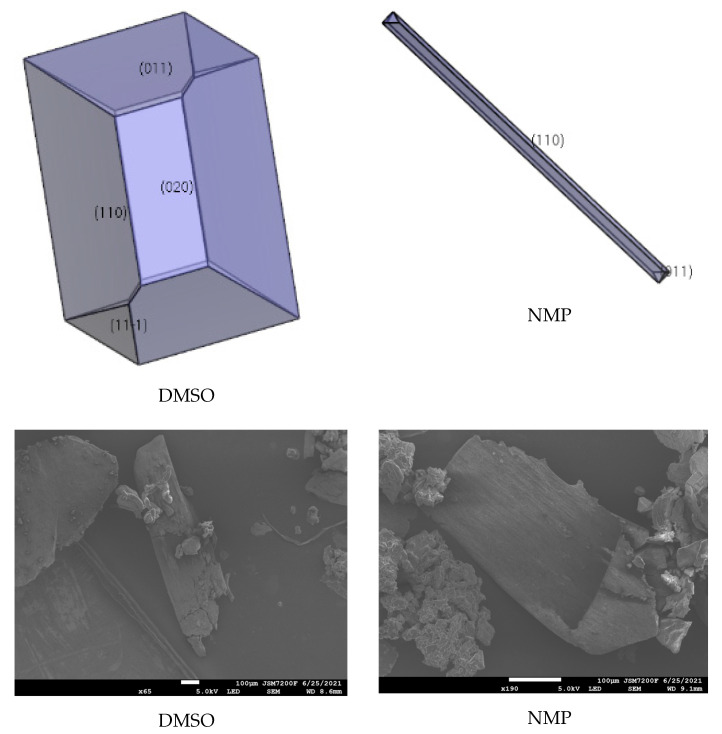
Crystal growth morphology of HATO in solvent by MAE model and recrystallization experiment.

**Figure 8:**
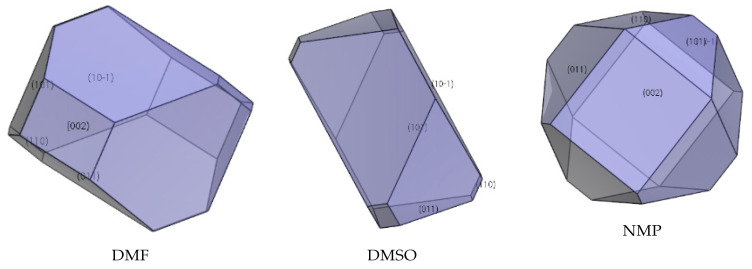

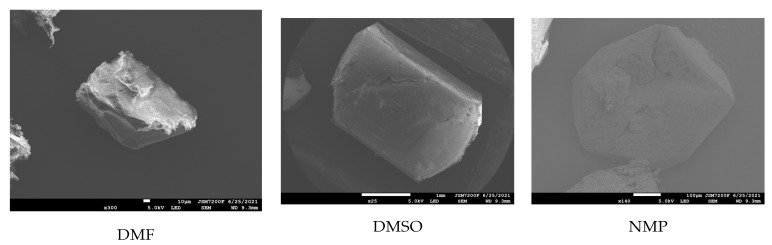
Crystal growth morphology of FOX-7 in solvent by MAE model and recrystallization experiment.

The interaction of solvents in the morphologically important crystal facets was simulated by the molecular dynamic method (shown in Table 4, Table 5 and Table 6). The interaction energy (*E*_int_) is the energy between each crystal face and solvent molecule which is negative value in three crystals, indicating that the adsorption of solvent in the crystal face was exothermic, during which the solvent will spontaneously adhere to the crystal interface [30]. In Table 4, Table 5 and Table 6, we can see that different solvents have different effects on each crystal facet, which leads to the change of the importance of each crystal facet during the process of crystal growth. The crystal morphologies of CL-20, HATO, and FOX-7 in different solvents predicted and experimented were shown in Figure 6, Figure 7 and Figure 8, respectively. Compared with the morphology in vacuum, the crystal morphology predicted by MAE model in solvent is very obviously different.

In Table 4, we can see the change of the important crystal facet of CL-20. In ethyl acetate, the {0 0 2} facet is the most important crystal facet, which possesses the largest area percentage. The cohesive energy density (CED) of {0 0 2} is −3886.14 kcal/mol and the attachment energy of {0 0 2} varied from −92.86 kcal/mol to −47.13 kcal/mol. Furthermore, the crystal facets of {0 1 1}, {1 1 −1}, and {1 0 1} tend to disappear in ethyl acetate. In DMF and acetone, the most important crystal facet is still the {0 1 1} facet, which is in agreement with in-vacuum. In DMF and acetone, the CED of {0 1 1} are −8076.94 kcal/mol and −5979.91 kcal/mol, respectively. The attachment energy varied from −84.78 kcal/mol to 249.90 kcal/mol and from −84.78 kcal/mol to 6741.06 kcal/mol, respectively. However, in acetone, the crystal facets of {1 1 −1}, {0 0 2}, and {1 0 1} tend to disappear, which is different from in DMF solution. In acetonitrile, the most important crystal facet is {0 0 2}, which possesses the CED with −4189.15 kcal/mol. The crystal facets of {1 1 0} and {1 0 1} tend to disappear.

The important crystal facet of HATO in solvent is shown in Table 5. we can see that in DMSO, the {1 1 0} facet is the most important crystal facet, which possesses the largest area percentage. The CED of {1 1 0} is −2633.03 kcal/mol and the attachment energy of {1 1 0} varied from −26.62 kcal/mol to 321.27 kcal/mol. Furthermore, the crystal facets of {1 0 0} and {1 1 −1} tend to disappear in DMSO. In NMP, the most important crystal facet is also the {1 1 0} facet, which is agree with in DMSO. The CED of {1 1 0} is −3031.07 kcal/mol and the attachment energy varied from −26.62 kcal/mol to −11.94 kcal/mol. In DMSO, the crystal facet of {1 0 0} tends to disappear, however, in NMP solution, there are three crystal facets tend to disappear, involving {1 0 0}, {0 2 0}, and {1 1 −1}.

The important crystal facet of FOX-7 in solvent is shown in Table 6. we can see that in DMF, the {0 1 1} facet is the most important crystal facet, which possesses the largest area percentage. The CED of {0 1 1} is −4643.31 kcal/mol and the attachment energy of {0 1 1} varied from −37.04 kcal/mol to 145.83 kcal/mol. In DMSO, the {1 0 −1} facet is the most important crystal facet. The CED of {1 0 −1} is −3842.51 kcal/mol and the attachment energy of {1 0 −1} varied from −36.61 kcal/mol to 125.28 kcal/mol. Furthermore, the crystal facets of {0 0 2} tend to disappear in DMSO. In NMP, the most important crystal facet is the {1 1 0} facet. The CED of {1 1 0} is −4630.11 kcal/mol and the attachment energy varied from −46.51 kcal/mol to 165.88 kcal/mol.

In Figure 6, we can see that the morphology of CL-20 in ethyl acetate and DMF is a column, whereas, in acetonitrile and acetone the morphology tends to a rhombus. Meanwhile, in Figure 7, the morphology of HATO is rodlike in DMSO and NMP. In Figure 8, the morphology of FOX-7 is irregular polyhedron in NMP, DMSO, and NMP. Moreover, we can know that the morphology predicted of three crystals in different solvents is quite consistent with the corresponding experimental results.

## 3. Experimental Computational Details

### 3.1. Hirshfeld Surface

The Hirshfeld surface (HS) and corresponding 2D fingerprints were calculated by CrystalExplorer [31]. In CrystalExplorer, the electron density, which comes from tabulations of atomic wavefunctions, was used to compute the HS. The pro-molecule electron density $\rho_{\mathrm{promol}}\left(r\right)$ and the pro-crystal electron density $\rho_{\mathrm{procryst}}\left(r\right)$ were constructed based on the spherical atomic electron densities $\rho_{A}\left(r\right)$. Then, the weight function was defined based on $\rho_{\mathrm{promol}}\left(r\right)$ and $\rho_{\mathrm{procryst}}\left(r\right)$. The weight function is as follows:

(1) Wr=∑A∈moleculeρAr∑A∈crystalρAr

In all space, this continuous scalar function has 0 < $W\left(r\right)$ < 1, and the 0.5 isosurface of this function is the HS. The 2D fingerprint was displayed to its HS by CrystalExplorer.

### 3.2. Reduced Density Gradient

Reduced density gradient (RDG) is powerful to reveal the noncovalent interaction (NCI) [32,33,34]. RDG is defined as follows:

(2) RDGr=123π213∇ργργ43

where $\rho \left(\gamma \right)$ is the electron density. The NCI plots were calculated by Multiwfn [35] and visualized using the VMD program [36]. 

### 3.3. Electrostatic Potential Surface

The calculation function of the electrostatic potential surface (ESP) is as follows:

(3) $$V_{tot}\left(r\right)=V_{nuc}\left(r\right)+V_{ele}\left(r\right)=\underset{A}{\sum }\frac{Z_{A}}{\left|R_{A}-r\right|}-\int \frac{\rho \left(r^{\prime }\right)}{\left|r-r^{\prime }\right|}dr$$

The electrostatic interaction between a unit point charge placed at r and the system of interest can be measured by this function. ESP was calculated by Multiwfn [35] and visualized using the VMD program [36]. The cartesian atomic coordinates can be found in Supplementary Materials: Tables S1–S3.

### 3.4. Model and Computational Details

#### 3.4.1. Construction of Models

**Figure 9:**
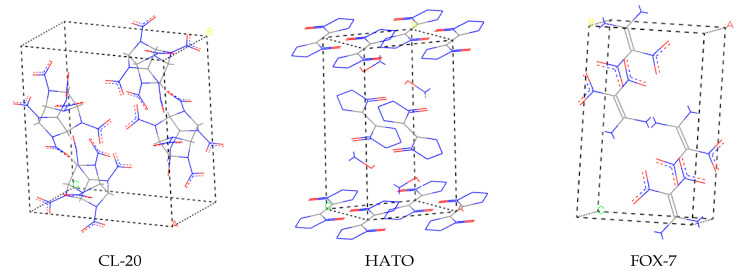
The crystal cell model of CL-20, HATO, and FOX-7, respectively.

All computation simulations were performed using the Materials Studio software. We constructed the initial unit cell structure of CL-20, HATO, and FOX-7 based on the Cambridge Structural Database available at Cambridge Crystallographic Data Centre (CCDC) using Material Studio software. The crystal cell model of three crystals is shown in Figure 9.

#### 3.4.2. Choice of Force Field

The force field plays a very important role to obtain reliable molecular dynamics simulation result. The popular COMPASS (condensed-phase optimized molecular potentials for atomistic molecular dynamics studies) force field has proved to be a universal all-atom force field, which has been calibrated from empirical parametrization techniques and state-of-the-art ab initio methods [8]. The COMPASS is suitable for molecular dynamic simulations involving conformation, vibration, structure, energy, and physical properties simulation of nitro (−NO_2_) containing compounds, such as RDX, HMX, and CL-20 [37,38]. So, CL-20 and FOX-7 were calculated by the COMPASS force field. However, there was no reasonable parameter for the N-oxides on the azole ring of HATO based on COMPASS force field in the simulation process. This result can be calibrated when replace COMPASS force field with Dreiding force field. Dreiding force field is a purely diagonal forcefield with harmonic valence terms and a cosine Fourier expansion torsion term and is more suitable for the N-oxides on the azole ring [39]. So, HATO cell structure was calculated by the Dreiding force field.

#### 3.4.3. Structure Optimization and Morphology in Vacuum

The atomic positions, lattice parameters and the geometry structure of CL-20, HATO, and FOX-7 were firstly optimized to minimize the total energy and obtain an optimized molecule geometry by the selected force field. Then, the crystal morphology of CL-20, HATO, and FOX-7 was simulated under vacuum with an attachment energy (AE) model, in which the growth morphology method was employed to identify the morphologically important facets of these crystals.

#### 3.4.4. Construction of Interface Models

The morphologically important growth crystal facets of CL-20, HATO, and FOX-7 crystals were cut to obtain the 3D periodic structure. Next, the periodic supercell was constructed. In the Amorphous Cell module, a solvent box was built with 200 molecules randomly distributed solvent molecules and it was consistent with the 3D periodic structure sizes of the important facets. Due to a favourable solubleness of CL-20 in ethyl acetate, N,N-Dimethylformamide(DMF), acetonitrile and acetone, these solvents were choice during the computation [40]. In the same way, the solvent molecules of HATO were dimethyl sulfoxide (DMSO) and N-methylpyrrolidone (NMP), and the solvent molecules of FOX-7 were DMF, DMSO, and NMP. Then, optimizing the solvent layer by the selected force field. The two-layer structure model containing solvent layer and main facet was constructed, which has a 50 Å thickness of the vacuum layer to eliminate the effect of additional free boundaries with a periodic boundary.

#### 3.4.5. Molecular Dynamic Simulation

The ensemble with constant temperature and constant volume (NVT)-MD simulation was carried out on CL-20, HATO, and FOX-7 two-layer structure models at room temperature (298 K) to obtain their equilibrium structure. The Berendsen thermostat was used to control the temperature. The total simulation time was set as 300 ps with a time step of 1 fs and every 1000 time steps output a frame. The calculations of the Van der Waals (vdW) and electrostatic force were performed by an atom-based method and the Ewald method, respectively. The cut-off distance of the atom-based method was set to 12.5 Å, and the Ewald accuracy of the Ewald method was set to 0.001 kcal/mol. Due to a reduction in the attachment energy in a vacuum, a modified attachment energy (MAE) model is more suitable for solvent screening. So, CL-20, HATO, and FOX-7 two-layer structure models were calculated with the MAE model. Eventually, the crystal morphologies of the CL-20, HATO, and FOX-7 were predicted by the MAE model in different solvent medium.

## 4. Conclusions

In this work, we investigated the intermolecular interaction, crystal morphology change, and energy of CL-20, HATO, and FOX-7 with quantum chemical computation. Compared with HATO and FOX-7, more red dots in Hirshfeld surfaces of CL-20 indicated more intermolecular interactions, especially hydrogen bonds. Noncovalent interaction analysis showed that apart from hydrogen bond, there were Van der Waals interactions exist in CL-20, HATO, and FOX-7. The ESP analysis detected the reactive site between solvent and crystal face. MD simulation manifested the crystal habit in vacuum by AE model have apparently change compared with in the solvent system. Furthermore, different solvents also have different effects on crystal morphology. These results indicate that solvent and environmental condition can enormously affect crystal morphology. Our findings give us a better understanding of the crystal engineering of energetic materials.

## Figures and Tables

**Table 1:** Dominant crystal facets of CL-20.

Facet	Multiplicity	*d*_hkl_/Å	Surface Area	*E*_att_(Total) /(kcal/mol)	*E*_att_(vdW) /(kcal/mol)	*E*_att_(Electrostatic) /(kcal/mol)	Distance/Å	Total Facet Area (%)
{0 1 1}	4	8.81	153.78	−84.78	−33.51	−51.27	84.78	39.67
{1 0 −1}	2	8.06	168.01	−87.92	−34.69	−53.23	87.92	13.14
{1 1 0}	4	6.94	195.31	−91.04	−38.95	−52.09	91.04	26.12
{1 1 −1}	4	6.73	201.15	−95.97	−38.15	−57.81	95.97	8.10
{0 0 2}	2	6.33	106.85	−92.86	−37.69	−55.17	92.86	10.27
{1 0 1}	2	2.29	215.46	−110.24	−47.12	−63.12	110.24	2.71

**Table 2:** Dominant crystal facets of HATO.

Facet	Multiplicity	*d*_hkl_/Å	Surface Area	*E*_att_(Total) /(kcal/mol)	*E*_att_(vdW) /(kcal/mol)	*E*_att_(Electrostatic) /(kcal/mol)	Distance/Å	Total Facet Area (%)
{1 0 0}	2	6.59	70.49	−20.11	−10.33	−9.78	20.11	29.59
{1 1 0}	4	5.66	82.11	−26.62	−14.76	−11.86	26.62	8.60
{0 2 0}	2	5.52	42.11	−24.39	−13.57	−10.82	24.39	21.84
{0 1 1}	4	5.50	84.57	−29.70	−11.56	−18.14	29.70	27.67
{1 1 −1}	4	4.47	104.06	−30.39	−18.29	−12.10	30.39	12.30

**Table 3:** Dominant crystal facets of FOX-7.

Facet	Multiplicity	*d*_hkl_/Å	Surface Area	*E*_att_(Total) /(kcal/mol)	*E*_att_(vdW) /(kcal/mol)	*E*_att_(Electrostatic) /(kcal/mol)	Distance/Å	Total Facet Area (%)
{0 1 1}	4	5.79	86.64	−37.04	−22.26	−14.78	37.04	50.25
{1 0 −1}	2	5.75	87.11	−36.61	−21.04	−15.57	36.61	24.61
{0 0 2}	2	5.68	44.15	−63.85	−22.94	−40.91	63.85	1.75
{1 0 1}	2	5.61	89.29	−49.43	−17.38	−32.05	49.43	12.85
{1 1 0}	4	4.70	106.72	−46.51	−25.10	−21.41	46.51	10.53

**Table 4:** Adsorption energies between the solvent and different CL-20 crystal faces.

	{*h l k*}	*E*_int_/(kcal/mol)	*E*_s_/(kcal/mol)	*E*_att_ */(kcal/mol)	CED/(kcal/mol)	Total Facet Area (%)
ethyl acetate	{0 1 1}	−8454.70	−6825.84	6741.06	−6205.59	−
	{1 0 −1}	−178.58	−148.39	60.47	−6029.59	36.38
	{1 1 0}	−560.59	−326.85	235.81	−6049.49	11.97
	{1 1 −1}	−724.11	−449.72	353.76	−5684.58	−
	{0 0 2}	−44.07	−45.73	−47.13	−3886.14	51.66
	{1 0 1}	−671.64	−375.71	265.38	−5984.99	−
DMF	{0 1 1}	−799.08	−334.68	249.90	−8076.94	48.38
	{1 0 −1}	−778.27	−425.29	337.37	−6253.02	14.74
	{1 1 0}	−896.18	−486.36	395.32	−6422.75	4.99
	{1 1 −1}	−975.45	−502.34	406.37	−5620.82	1.18
	{0 0 2}	−553.03	−328.25	235.40	−3980.93	14.17
	{1 0 1}	−864.72	−388.23	277.99	−5809.20	16.54
acetonitrile	{0 1 1}	−881.48	−456.95	372.18	−6475.99	4.11
	{1 0 −1}	−758.41	−335.41	267.48	−6367.03	8.86
	{1 1 0}	−1354.59	−509.52	418.48	−6424.70	−
	{1 1 −1}	−930.51	−389.32	293.35	−6151.77	28.51
	{0 0 2}	−340.98	−193.02	100.17	−4189.15	58.53
	{1 0 1}	−1147.79	−426.61	316.38	−6287.97	−
acetone	{0 1 1}	−8454.70	−6825.84	6741.06	−5979.91	48.96
	{1 0 −1}	−178.58	−148.39	60.47	−5807.82	6.38
	{1 1 0}	−560.59	−326.85	235.81	−5854.99	44.66
	{1 1 −1}	−724.11	−449.72	353.76	−5391.30	−
	{0 0 2}	−44.07	−45.73	−47.13	−3798.27	−
	{1 0 1}	−671.64	−375.71	265.48	−5671.363	−

*E*_att_ * is the modified attachment energy.

**Table 5:** Adsorption energies between the solvent and different HATO crystal faces.

	{*h l k* }	*E*_int_/(kcal/mol)	*E*_s_/(kcal/mol)	*E*_att_ */(kcal/mol)	CED/(kcal/mol)	Total Facet Area (%)
DMSO	{1 0 0}	−258.23	−461.19	441.08	−2648.26	−
	{1 1 0}	−222.28	−347.89	321.27	−2633.03	53.62
	{0 2 0}	−224.44	−463.73	439.34	−2025.01	8.95
	{0 1 1}	−312.43	−486.03	456.32	−2665.47	29.59
	{1 1 −1}	−340.49	−510.72	480.34	−2633.51	7.84
NMP	{1 0 0}	−163.59	−357.24	337.13	−2996.00	−
	{1 1 0}	−13.64	−14.69	−11.94	−3031.07	97.97
	{0 2 0}	−90.73	−278.65	254.26	−2249.33	−
	{0 1 1}	−188.83	−322.94	293.24	−3104.75	2.03
	{1 1 −1}	−246.51	−339.18	308.79	−3017.11	−

*E*_att_ * is the modified attachment energy.

**Table 6:** Adsorption energies between the solvent and different FOX-7 crystal faces.

	{*h l k*}	*E*_int_/(kcal/mol)	*E*_s_/(kcal/mol)	*E*_att_ */(kcal/mol)	CED/(kcal/mol)	Total Facet Area (%)
DMF	{0 1 1}	−256.06	−182.86	145.83	−4643.31	46.35
	{1 0 −1}	−225.41	−162.57	125.96	−4609.75	31.50
	{0 0 2}	−253.23	−244.83	180.99	−3238.98	9.19
	{1 0 1}	−332.22	−243.21	193.77	−4551.62	10.93
	{1 1 0}	−355.10	−254.96	208.45	−4537.00	2.04
DMSO	{0 1 1}	−243.04	−252.54	215.50	−3915.75	29.29
	{1 0 −1}	−158.55	−161.89	125.28	−3842.51	36.76
	{0 0 2}	−222.09	−402.84	338.99	−2642.95	−
	{1 0 1}	−202.64	−190.50	141.07	−3834.91	31.15
	{1 1 0}	−336.46	−289.11	242.60	−3635.65	2.81
NMP	{0 1 1}	−231.32	−204.53	167.49	−4817.41	26.78
	{1 0 −1}	−227.52	−200.47	163.86	−4750.23	14.81
	{0 0 2}	−133.69	−216.62	152.78	−3403.93	21.33
	{1 0 1}	−251.54	−236.91	187.48	−4556.55	8.65
	{1 1 0}	−311.19	−212.39	165.88	−4630.11	28.44

*E*_att_ * is the modified attachment energy.

## Data Availability

Not applicable.

## Supplementary Materials

The following supporting information can be downloaded at: https://www.mdpi.com/article/10.3390/molecules27123755/s1, Table S1: Cartesian atomic coordinates for CL-20 used for reduced density gradient analysis; Table S2: Cartesian atomic coordinates for HATO used for reduced density gradient analysis; Table S3. Cartesian atomic coordinates for FOX-7 used for reduced density gradient analysis.

## Sample Availability

Samples of the compounds are not available from the authors.
