# Supplementary material for: Noncovalent Interactions and Crystal Structure Prediction of Energetic Materials
Source: Molecules. 2022 Jun 10;27(12):3755. doi: 10.3390/molecules27123755 (PMC9229783; doi:10.3390/molecules27123755)
Supplement: Supplementary file 1 [file molecules-27-03755-s001.zip › molecules-1754510-supplementary.pdf]

Supplementary Materials

Noncovalent Interactions and Crystal Structure Prediction of Energetic Materials

Yan Liu [1,2], Chongwei An [1,*], Ning Liu [3], Minchang Wang [3], Baoyun Ye [1] and Dongjie Liao [1]

[1] School of Environment and Safety Engineering, North University of China, Taiyuan 030051, China; 18234159991@sina.cn (Y.L.); 18334788650@163.com (B.Y.); liaodongjie163@163.com (D.L.)

[2] Department of Environmental and Safety Engineering, Taiyuan Institute of Technology, Taiyuan 030008, China;

[3] Xi'an Modern Chemistry Research Institute, Xi'an 710065, China; flackliu@sina.com (N.L.); wmc204@163.com (M.W.)

* Correspondence: anchongwei@yeah.net

Reduced density gradient analysis

Table S1. Cartesian atomic coordinates for CL-20 used for reduced density gradient analysis.

atom		location	
C	0.13400567	-0.77768735	-1.28540858
H	0.19456154	-1.10075800	-2.20480801
N	-0.97243569	-1.36494233	-0.56088132
N	-1.53667993	-2.53298205	-1.10759444
O	-2.20774124	-3.17806075	-0.33977572
O	-1.27751431	-2.77405139	-2.25897407
C	-0.63238851	-1.32649284	0.87456595
H	-0.92199663	-2.18361129	1.29966197
N	-1.26332072	-0.20112863	1.48819936
N	-2.59287433	-0.39087625	1.87820934
O	-3.28072537	0.59115555	1.81038892
O	-2.90048400	-1.49779969	2.22900252
C	-0.86696114	1.05678707	0.93617700
H	-1.34052821	1.76453360	1.43080320
N	1.28678338	1.12488266	-0.49287992
N	-2.02594981	2.23738893	-0.92598398
O	-2.70220129	2.80756894	-0.10124801
O	-1.96928392	2.46051598	-2.11214008
C	-0.05106137	0.84275022	-1.26241795
H	-0.09705799	1.19529340	-2.17158309
N	0.98726324	1.44145925	-0.46966227
N	1.39562562	2.72168665	-0.86628856
O	1.21249113	2.97810425	-2.03371284
O	2.04458637	3.35636544	-0.07920355
C	0.59706751	1.26220522	0.93754757
H	0.84188872	2.06020662	1.45113314
N	1.23947388	0.08720735	1.52431835
N	2.62481970	0.22789547	1.74708080
O	3.23726616	-0.75835280	2.07021284
O	3.07368266	1.34340535	1.59303995
C	0.91728166	-1.17979062	0.90028185
H	1.37181317	-1.91692289	1.37596880
N	1.33336104	-1.11716990	-0.51113548
N	2.05824602	-2.23070224	-0.99931737
O	2.88198767	-2.66036718	-0.23369579
O	1.84274176	-2.55653361	-2.13315741

Table S2. Cartesian atomic coordinates for HATO used for reduced density gradient analysis.

atom		location	
O	-1.36371517	-1.28992881	-0.00013706
N	-0.96609827	-0.01062006	0.00028470
N	-1.85348353	1.03283334	0.00067682
N	-1.12625852	2.17017072	0.00103719
N	0.19400273	1.86762844	0.00081917
C	0.29549628	0.50840916	0.00041170
O	3.14825590	1.55519690	-0.00122875
N	2.79077815	0.32540432	-0.00055087

N	3.74346918	-0.76542896	-0.00040641
N	3.04946274	-1.84254544	0.00030110
N	1.61501510	-1.57037847	0.00057204
C	1.50205216	-0.25560055	0.00019050
O	-4.37623658	0.11582208	-0.00208919
N	-3.90645842	-1.27516212	0.00046303
H	-4.29352776	-1.71604033	0.83771563
H	-2.74233515	-1.31491339	0.00146688
H	-4.29173862	-1.71826446	-0.83638581
H	-3.50712169	0.66033171	-0.00115361

Table S3. Cartesian atomic coordinates for FOX-7 used for reduced density gradient analysis.

atom		location	
O	0.18077118	2.30509821	0.01668020
O	2.01377820	1.18387852	-0.29077337
O	1.87837656	-1.21093006	0.64203276
O	0.33063788	-2.19510555	-0.49645822
N	-2.03673973	-1.18629209	0.02938151
H	-1.63398293	-1.93232036	-0.03538502
H	-2.87358381	-1.13988537	0.16929968
N	-2.11494525	1.08383174	0.10656995
H	-2.96038158	1.01270291	0.15954429
H	-1.65169396	1.79187460	0.18612116
N	0.78406341	1.21704893	-0.08401271
N	0.80073937	-1.17799883	0.03204380
C	-1.40301082	-0.02012726	0.02139252
C	0.04622892	0.02812260	-0.02794320
